# Reductions in 28-Day Mortality Following Hospital Admission for Upper Gastrointestinal Hemorrhage

**DOI:** 10.1053/j.gastro.2011.03.048

**Published:** 2011-07

**Authors:** Colin Crooks, Tim Card, Joe West

**Affiliations:** ⁎ Division of Epidemiology and Public Health, The University of Nottingham, Nottingham City Hospital, Nottingham, United Kingdom; ‡ Nottingham Digestive Diseases Centre, National Institute for Health Research Biomedical Research Unit, Queen's Medical Centre, Nottingham University Hospitals National Health Service Trust, Nottingham, United Kingdom; § Sherwood Forest Hospitals National Health Service Trust, Mansfield, United Kingdom

**Keywords:** Hospital Episode Statistics, Time Trends, Charlson Index, Bleeding, HES, Hospital Episodes Statistics, NHS, National Health Service, ICD, International Classification of Diseases, ONS, Office of National Statistics, BSG, British Society of Gastroenterology

## Abstract

**Background & Aims:**

It is unclear whether mortality from upper gastrointestinal hemorrhage is changing: any differences observed might result from changes in age or comorbidity of patient populations. We estimated trends in 28-day mortality in England following hospital admission for gastrointestinal hemorrhage.

**Methods:**

We used a case-control study design to analyze data from all adults administered to a National Health Service hospital, for upper gastrointestinal hemorrhage, from 1999 to 2007 (n = 516,153). Cases were deaths within 28 days of admission (n = 74,992), and controls were survivors to 28 days. The 28-day mortality was derived from the linked national death register. A logistic regression model was used to adjust trends in nonvariceal and variceal hemorrhage mortality for age, sex, and comorbidities and to investigate potential interactions.

**Results:**

During the study period, the unadjusted, overall, 28-day mortality following nonvariceal hemorrhage was reduced from 14.7% to 13.1% (unadjusted odds ratio, 0.87; 95% confidence interval: 0.84–0.90). The mortality following variceal hemorrhage was reduced from 24.6% to 20.9% (unadjusted odds ratio, 0.8; 95% confidence interval: 0.69–0.95). Adjustments for age and comorbidity partly accounted for the observed trends in mortality. Different mortality trends were identified for different age groups following nonvariceal hemorrhage.

**Conclusions:**

The 28-day mortality in England following both nonvariceal and variceal upper gastrointestinal hemorrhage decreased from 1999 to 2007, and the reduction had been partly obscured by changes in patient age and comorbidities. Our findings indicate that the overall management of bleeding has improved within the first 4 weeks of admission.

Gastrointestinal hemorrhage is the commonest cause of acute hospital admission to gastroenterology and therefore has a large impact on the acute medical admission workload. Changes in management have been shown in randomized controlled trials to improve outcome from gastrointestinal hemorrhage, but the largest observational studies of mortality trends following upper gastrointestinal hemorrhage report no improvement in overall mortality over the last 2 decades.^1–3^ This failure to demonstrate an improvement suggests either that clinical guidelines^4,5^ derived from the results of randomized controlled trials are not generalizable to the clinical population, that they are not being implemented appropriately, or that the patients have changed at the same time as the treatments. This latter explanation, with increasing age and comorbidity confounding the effects of therapy, has been proposed as the likely explanation.^6,7^ However, this has not been proven because to reliably measure the effect of changes in age and comorbidity on mortality necessitates larger studies than have been published. Therefore, we aimed to investigate current trends in mortality following admission from upper gastrointestinal hemorrhage in England and investigate whether these can be explained by population changes in age and comorbidity.

## Patients and Methods

### Database

The Hospital Episodes Statistics database (HES) contains information on all admissions to an NHS hospital in England, with over 12 million new records added each year. It is managed by the NHS information center and is available for research with ethical approval. All NHS hospitals within England are required to contribute to the database. There are currently 168 acute trusts in England; however, each of these trusts can manage more than 1 hospital, and over time trusts can merge and split. Over the course of our study, approximately 150–200 providers were contributing to the database. The available data consist of a number of records for each admission, which are called *episodes*. Each episode represents the time period of the admission that a patient was under the clinical care of a particular consultant team during their inpatient stay. A unique patient identifier allows all records for each patient to be identified and linked together. Each episode's time span is defined with a start and finish date as well as being assigned an admission and discharge date for the whole period of the inpatient stay. Each episode will have up to 14 diagnoses coded using International Classification of Diseases 10th revision (ICD-10); and up to 12 procedures coded using the United Kingdom Tabular List of the Classification of Surgical Operations and Procedures (OPCS) (version OPCS4). This database has been linked to the Office of National Statistics (ONS) death register since 1998.

### Study Population

#### Inclusion criteria

All admissions older than 15 years (chosen to be consistent with the lower age limit of previous British Society of Gastroenterology (BSG) audits of mortality in gastrointestinal hemorrhage^8,9^), which had an ICD-10 code for upper gastrointestinal hemorrhage, with a date of hemorrhage between January 1, 1999, and December 31, 2007, were extracted. Data were available for 2008 to allow complete follow-up of mortality for admissions occurring in December 2007. Upper gastrointestinal hemorrhage was defined as an ICD-10 code that specifically implied either variceal gastrointestinal hemorrhage: esophageal varices with hemorrhage (I85.0) or nonvariceal hemorrhage: Mallory–Weiss syndrome (K22.6), esophageal hemorrhage (K22.8) acute, or chronic gastric ulcer with hemorrhage including perforation with hemorrhage (K25.0, K25.2, K25.4, K25.6), acute or chronic duodenal ulcer with hemorrhage including perforation with hemorrhage (K26.0, K26.2, K26.4, K26.6), acute or chronic peptic ulcer with hemorrhage including perforation with hemorrhage (K27.0, K27.2, K27.4, K27.6), acute or chronic gastrojejunal ulcer with hemorrhage including perforation with hemorrhage (K28.0, K28.2, K28.4, K28.6), hematemesis (K92.0), melena (K92.1), or unspecified gastrointestinal hemorrhage (K92.2). This ICD-10 code list has previously been used in hospital data.^10^ Subsequent readmissions with upper gastrointestinal hemorrhage were included in the study and recorded as a readmission. We performed 2 sensitivity analyses to assess the affect of inaccuracies in coding. First, to assess the effect of under-reporting, we expanded the definition for variceal hemorrhage to include all admissions coded for esophageal hemorrhage (K22.8) and then reassessed the trends in mortality. Second, to assess whether there was over-reporting of cases that might not be a genuine upper gastrointestinal hemorrhage, we analyzed separately those who had and those who did not have an intervention of upper gastrointestinal endoscopy recorded (as defined by an OPCS4 code for an endoscopic procedure of the upper gastrointestinal tract).

#### Exclusion criteria

The study population was geographically limited to patients who were residents within England at the time of hospital admission. Admissions were excluded if they were coded with unspecified gastrointestinal hemorrhage (K92.2) and had a lower gastrointestinal endoscopy/diagnosis code but no upper gastrointestinal endoscopy code. Admissions were also excluded with the following: day case admission codes with no overnight stay (a majority of these admissions were for an outpatient endoscopy and would not have represented an acute presentation of hemorrhage but either a complication of endoscopy or a follow-up endoscopy to a previous bleed), invalid date codes as flagged by HES, date codes that were out of chronological order, invalid date of birth codes, invalid sex codes, or duplicate records for 1 episode.

### Outcome

Short-term mortality was defined as a date of death within 28 days of the start of the recorded episode of upper gastrointestinal hemorrhage. This included deaths that occurred after discharge from hospital but within the 28 days. The date and fact of death were obtained from the ONS death register using a probability matching algorithm based on NHS number, date of birth, postcode, and sex.^11^

### Exposures

The exposure of interest was defined as the year of upper gastrointestinal hemorrhage. Charlson index,^12^ sex, and age were assessed as potential confounders. The Charlson index was calculated for each upper gastrointestinal hemorrhage admission based on the diagnoses coded for all admissions up to and including the first upper gastrointestinal hemorrhage admission for each patient. The Charlson index is a validated comorbidity score that has been weighted to predict 1-year mortality. For analysis and reporting, it is combined into 3 groups: no comorbidity (0), a single comorbidity (1), and multiple or serious comorbidity (2). For analysis of variceal hemorrhage, the comorbidity of liver disease was excluded from the calculation of Charlson index because most variceal patients will have liver disease. The Charlson index has been adapted and validated for ICD-10 coding in administrative data^13,14^ and has previously been used in HES.^15^ As a sensitivity analysis, we also assessed the use of an alternative measure of comorbidity called the *Elixhauser index*^16^ that was derived to predict mortality during the inpatient stay. Although it has the potential to be a more appropriate measure for our study than the Charlson index, it has not been previously validated within HES, so it was not used for our primary analysis. The recorded age was grouped into age bands of 15–29 years, 30–59 years, 60–79 years, and older than 80 years. A further analysis assessed whether using a higher minimum age limit of 18 years altered the results. We calculated the length of inpatient stay as the number of days between admission and discharge dates. We defined admissions as either having a higher probability of being an acute bleed on admission (if an upper gastrointestinal hemorrhage was coded on the first episode in a nonelective admission) or as lower probability of being an acute bleed on admission with a higher probability of being an inpatient bleed (if the coding occurred after the first episode within a nonelective admission, or during an elective [nonemergency] admission). Hereafter, these are referred to, respectively, as acute admissions and inpatient bleeds. To assess trends in diagnoses that were associated with a gastrointestinal hemorrhage code, we extracted additional diagnoses for gastritis/duodenitis, Mallory–Weiss syndrome, any peptic ulcer, gastric ulcer, duodenal ulcer, and malignancy.

### Statistical Analysis

We analyzed variceal and nonvariceal hemorrhage admissions separately. After the exclusions described above, 28-day case fatalities were calculated by age group, sex, year, grouped Charlson index, and acute or inpatient hemorrhage. A case-control study analysis was carried out with cases defined as patients who had died by 28 days and controls as patients who were alive at 28 days. The primary exposure of interest was defined as year of upper gastrointestinal hemorrhage. A logistic regression model was constructed to adjust for the change in mortality over the study period by sex, age group, and Charlson index. Variables that changed the odds of mortality were judged to be confounders. We assessed whether there was a trend in mortality over time and whether this could be modelled as a linear trend using likelihood ratio tests. We also performed a secondary analysis comparing trends in mortality that occurred before discharge and trends in mortality that occurred after discharge. The calculation of postdischarge mortality excluded patients who had died as inpatients. In addition, to determine whether the changes in mortality varied for different ages, sex, and comorbidities, the model was also tested for interactions between each of the variables and year of bleed with likelihood ratio testing. If there was evidence against the null hypothesis of no interaction, stratified results were presented. The use of the a priori age groups was assessed against alternative groupings of 5-year age bands or age as a linear variable. All analysis was performed using Stata version 10 (StataCorp LP, College Station, TX).

## Results

### Study Population and Exclusions

**Figure 1:**
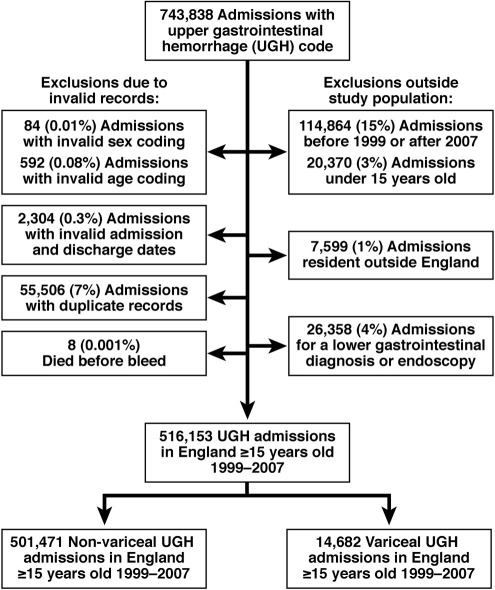
Flowchart of exclusions from study population.

There were 516,153 upper gastrointestinal hemorrhage admissions identified after exclusions (shown in Figure 1) of which 501,471 (97%) were nonvariceal bleeds, and 14,682 (3%) were variceal bleeds.

### Mortality Ascertainment

Seventy-four thousand nine hundred ninety-two deaths occurred within 28 days of the date of upper gastrointestinal hemorrhage, giving an overall case fatality rate of 14.5% (95% confidence interval [95% CI]: 14.4%–14.6%). Of these, 10,977 deaths (15%) occurred after discharge from hospital but within 28 days of hemorrhage. Only 312 (3%) of postdischarge deaths were coded as a subsequent hospital admission within the HES dataset.

### Univariable Analysis

**Figure 2:**
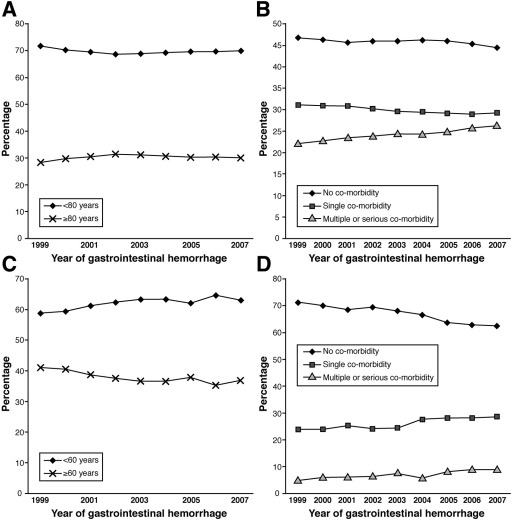
Trends in age and comorbidity measured by grouped Charlson index (percentage of population shown). (*A*) Percentage of nonvariceal hemorrhage patients in each age band. (*B*) Percentage of nonvariceal hemorrhage patients in each comorbidity group. (*C*) Percentage of variceal hemorrhage patients in each age band. (*D*) Percentage of variceal hemorrhage patients in each comorbidity group.

The population characteristics for nonvariceal and variceal hemorrhage are shown in Table 1. The median age for nonvariceal bleeds was 71 years (interquartile range, 50–81 years) and, for variceal bleeds, was 55 years (interquartile range, 45–66 years). Forty-six percent of those presenting with nonvariceal hemorrhage had no comorbidity recorded, compared with 67% of those presenting with variceal hemorrhage after the exclusion of liver disease from the calculation of comorbidity. The population age structure and comorbidity varied over the study period (Figure 2) with a peak in the proportion of nonvariceal admissions over 80 years old in 2002. This matched the peak in case fatality in the same year (Table 1). There was a reduction over time in the proportion of those presenting with variceal hemorrhage who were less than 60 years old (Figure 2). The comorbidity for both groups increased over the study period. Median length of stay for nonvariceal hemorrhage was 4 days (interquartile range, 1–8 days) and for variceal hemorrhage was 7 days (interquartile range, 4–12 days). The length of stay reduced over the study period for nonvariceal hemorrhage from 4 (interquartile range, 2–8 days) to 3 (interquartile range, 1–6 days) (*P* < .001 nonparametric test for trend), but there was no reduction for variceal hemorrhage.

### Nonvariceal and Variceal Hemorrhage

The overall 28-day case fatality following a nonvariceal hemorrhage admission was 14% and, following a variceal hemorrhage admission, was 23% (Table 1). From 1999 to 2007, the unadjusted 28-day mortality following nonvariceal hemorrhage reduced from 14.7% to 13.1% (unadjusted odds ratio [OR], 0.87; 95% CI: 0.84–0.90). The unadjusted mortality following variceal hemorrhage reduced from 24.6% to 20.9% (unadjusted OR, 0.81; (95% CI: 0.69–0.95).

### Acute Hemorrhage on Admission Compared With Inpatient Hemorrhage

Twenty-eight-day mortality for an acute admission with hemorrhage reduced over the study period for nonvariceal hemorrhage from 11.3% to 9.3% (unadjusted OR, 0.81; 95% CI: 0.77–0.85) and, for variceal hemorrhage, from 21.3% to 17.3% (unadjusted OR, 0.77; 95% CI: 0.62–0.95). Twenty-eight-day mortality for cases with an inpatient hemorrhage also reduced over the study period, for nonvariceal hemorrhage from 20.0% to 18.4% (unadjusted OR, 0.91; 95% CI: 0.86–0.95) and, for variceal hemorrhage, from 32% to 29% (unadjusted OR, 0.88; 95% CI: 0.67–1.14).

### Multivariate Analysis

The odds of mortality for each year were altered when adjusted separately for each of the potential confounders of age, sex, and Charlson index. The slight peak in mortality in 2002 was removed when adjusting for the increase in age in 2002. The use of alternative groupings for age did not alter the estimates. An alternative minimum age limit of 18 years did not alter the findings of the analysis for mortality. Adjusting for increases in comorbidity had the largest effect on the reduction in mortality. The multivariate model adjusting for all these variables is shown in Table 2. Age and comorbidity were stronger confounders for nonvariceal than variceal hemorrhage.

There was evidence of a linear trend in mortality over time, for both nonvariceal hemorrhage and variceal hemorrhage (*P* < .001), and there was minimal evidence to suggest that a linear model was inappropriate for the data (test for departure from a linear trend; nonvariceal hemorrhage, *P* = .061; variceal hemorrhage, *P* = .94). The adjusted average annual reduction in odds of mortality for nonvariceal hemorrhage was 2.5% (average annual OR, 0.97; 95% CI: 0.97–0.98) and, for variceal hemorrhage, was 3.5% (average annual OR, 0.96; 95% CI: 0.95–0.98). Assessing age, sex, and comorbidity adjusted trends following the diagnoses of gastritis/duodenitis, Mallory–Weiss syndrome, any peptic ulcer, gastric ulcer, duodenal ulcer, or malignancy associated with nonvariceal hemorrhage found that there were similar reductions in mortality following all these diagnoses (see Table 3). A sensitivity analysis was conducted including esophageal hemorrhage codes (K22.8) as a variceal hemorrhage admission, and this estimated an annual reduction in odds of mortality of 3.6% (average annual OR, 0.96; 95% CI: 0.95–0.98). The second sensitivity analysis found a similar reduction in nonvariceal hemorrhage admissions who had an endoscopy recorded (average annual OR, 0.97; 95% CI: 0.96–0.97) to those who did not have an endoscopy recorded (average annual OR, 0.96; 95% CI: 0.96–0.97). This was also the case for variceal hemorrhage, although because only a few cases did not have an endoscopy, there was greater uncertainty (with endoscopy: average annual OR, 0.98; 95% CI: 0.96–0.99; without endoscopy: average annual OR, 0.95, 95% CI: 0.92–0.98). The third sensitivity analysis used the Elixhauser index to adjust for comorbidity, and this showed a slightly increased average annual reduction compared with using the Charlson index to adjust for comorbidity (nonvariceal hemorrhage OR, 0.96; 95% CI: 0.96–0.97). However, the overall model with the Elixhauser index did not have as good a fit to the data as when the Charlson index was used to adjust for comorbidity.

Reanalyzing the age, sex, and comorbidity adjusted trends for mortality only occurring before discharge demonstrated the same reduction in inpatient mortality as in the main analysis (nonvariceal average annual adjusted mortality OR, 0.97; 95% CI: 0.97–0.98). However, the mortality after discharge increased slightly (nonvariceal average annual adjusted mortality OR, 1.02; 95% CI: 1.02–1.03). Further analyses for interactions demonstrated different time trends for different ages and different levels of comorbidity for nonvariceal hemorrhage (likelihood ratio tests for interactions of both age and comorbidity with year, *P* < .001) but not for variceal hemorrhage (year and age, *P* = .29; year and comorbidity, *P* = .67). Consequently, the age-specific stratum average annual changes in odds of mortality for nonvariceal hemorrhage are presented in Table 4. The annual improvement in odds of mortality was minimal for those presenting 80 years and older compared with all the other age groups. Further stratifying the model by age and comorbidity (Table 5) demonstrated that, within each age-specific stratum, the improvement in mortality did not differ by the level of comorbidity. Therefore, the final model of a linear trend in 28-day mortality for nonvariceal hemorrhage is the model shown in Table 4, with confounding by comorbidity adjusted for by logistic regression and effect modification demonstrated by stratifying the results by age. The final model of a linear trend in 28-day mortality for variceal hemorrhage demonstrated only confounding by both comorbidity and age with no effect modification.

## Discussion

The failure of previous studies to demonstrate improvements in mortality after upper gastrointestinal hemorrhage at the population level calls into question the value of therapeutic changes that are of proven benefit to individuals. In an increasingly challenging economic environment, clinicians will need to be able to demonstrate that increased therapeutic expenditure really does bring benefits. That 28-day mortality for equivalent patients, following hospital admission for both nonvariceal and variceal upper gastrointestinal hemorrhage, has reduced by 2% and 3%, respectively, year on year in England over the period 1999 to 2007 is therefore of great importance. The demonstration that this can be shown through the analysis of routinely collected data may be of great value in the assessment of other conditions.

### Strengths and Limitations

When, as in this case, a study's findings differ from the previous literature, we must ask whether this is because the current or previous studies were in error or whether they are in reality observing different things. The data source chosen for our study provides key advantages. The study is the largest to date of mortality after hospital admission for gastrointestinal hemorrhage and therefore has power to demonstrate trends that would be missed in smaller studies. It also has power to demonstrate variations in trends between subgroups of the population such as the smaller reduction in mortality in those over 80 years old with nonvariceal hemorrhage. The provision within the dataset of information on the previously suggested confounders of age and comorbidity is also of great benefit and has allowed us to clearly show and correct for this confounding.

Another key advantage of the current study is the linkage of clinical data with the ONS death register, ensuring that almost all deaths are captured in the study population. Hospital admission data only capture deaths occurring before discharge, which we found to be 86% of the deaths occurring within 28 days. Studies without such linkage will have missed a proportion of these deaths because postdischarge deaths will have been difficult to capture. Furthermore, any change in this capture over time may have biased results. The linkage used in the current study, depending as it does on probability matching, still leaves potential for some underestimation of mortality, but the robustness of the linkage coupled with its uniform methodology throughout the study period mean that bias because of this is unlikely to have occurred. The reduction in length of stay over the course of the study further emphasises the importance of identifying deaths following discharge to accurately calculate trends in mortality. The slight increase in postdischarge mortality might imply that the observed earlier discharge of patients was inappropriate; however, if management in hospital was no longer of benefit to a patient who is dying, then discharge might well be the most appropriate decision. The observed trends might therefore indicate a shift of unavoidable in-hospital mortality into the postdischarge period.

Patients who died in the emergency department before admission for endoscopy were not included in our study because hospital admissions data contain information only on admitted patients. However, because acute admission to the hospital for all upper gastrointestinal hemorrhages was standard practice within England, the admissions data will have captured almost all other relevant bleed presentations. We excluded patients who had a nonspecific code for gastrointestinal hemorrhage with a colonoscopy but no gastroscopy, and it is possible that these could have had an upper gastrointestinal bleed if they had died before a planned gastroscopy. However, this would be unlikely because usual practice would be to perform a gastroscopy before colonoscopy because of the easier access and greater therapeutic potential of gastroscopy.

There have been concerns about the accuracy of routine hospital admissions coding, in particular the coding of specific operations and the ascertainment of death for generating mortality rates for specific hospitals. However, a systematic review found a 91% median accuracy in diagnostic coding prior to our study period, and the most recent audit of selected samples of UK hospital data confirmed accuracy approaching 90%.^17^ Other comparisons of procedure coding have reported similar or higher rates of coding in the HES database compared with specialist clinical databases,^18,19^ and, with specific regard to upper gastrointestinal hemorrhage, the incidence of peptic ulcer hemorrhage in the HES data from 1992 to 1995 has been shown to be comparable with the 1993 regional BSG audit (32 vs 29 per 100,000 per year, respectively). Furthermore, by choosing our study period, we have ensured no systematic changes in coding because the ICD-10 coding system has been in continuous use in HES from 1995 to present. This, of course, does not exclude variation in rates of coding over the study period affecting our estimates. For example, if the potential error in coding was systematically changing over time with increased coding of patients' comorbidity rather than patients having more comorbidity, then clearly that could bias our results. However, the different trends in comorbidity for variceal and nonvariceal bleed admissions and different trends in mortality in different age and comorbidity strata suggest that there was no systematic change in comorbidity coding over the time period of our study. Under-reporting of the comorbidities in the Charlson index may have resulted in incomplete adjustment for comorbidity. However, although the alternative Elixhauser index assessed almost twice the number of comorbidities, it did not alter the adjustment of comorbidity in the model. Comorbidity adjustment by either index increased the magnitude of the mortality reduction, and, therefore, any residual confounding in this regard would only, we believe, cause an underestimate of the real mortality trend in our study.

### Other Studies

A PubMed search, to October 2010, found the largest comparable population-based study for nonvariceal hemorrhage mortality trends used a Canadian hospital discharge database with ICD-10 and ICD-9 codes. However, it identified less than one-third of the number of bleeds used for this study (n = 142,363) and was not able to identify a reduction in case fatality for nonvariceal hemorrhage between 1993 and 2003.^3^ The researchers adjusted for changes in age but not for changes in comorbidity. They also only identified deaths that occurred before discharge. The low mortality identified in this study (3.5%) is similar to other North American^20^ and Mediterranean^1,21^ studies but is much lower than other European studies.^2,22,23^ However, a study of Medicare patients in the United States found that the proportion being managed as outpatients varied between states from 18.6% to 45.3%.^24^ These differences in practice would lead to differences in inpatient study populations and confound comparisons with countries such as England where outpatient management is not routine.

Although the most recent report from the US National Inpatient Sample showed a 23% reduction in upper gastrointestinal hemorrhage mortality from 1998 to 2006 (n = unreported because only extrapolated estimates from the 20% sample are provided),^20^ this was a global figure for the reduction seen at the end of the study rather than year on year, and it did not distinguish variceal and nonvariceal hemorrhage. Another report from the US National Inpatient Sample noted an adjusted reduction in variceal hemorrhage from 18% to 12%.^25^ However, in the study period of both these reports, the number of states in the sampling frame almost doubled from 22 to 40. The reports therefore compare different populations from each time period, and, although a number of weighting procedures are used, the estimates remain susceptible to selection bias.

One smaller study from Wales (n = 24,421) used the same ICD-10 definitions as our study and also found an overall reduction in case fatality but did not report variceal and nonvariceal hemorrhage mortality trends separately or trends in different age and comorbidity strata.^10^ Other nonvariceal hemorrhage studies from Spain (n = 17,663),^1^ The Netherlands (n = 1720),^2^ Greece (n = 1304),^21^ France (n = 1165),^23^ and Italy (n = 1126)^22^ did not identify reductions in nonvariceal inpatient mortality. Although these were large studies, they may have been underpowered to detect a change, and none of them adjusted the trends in case fatality for changes in comorbidity. Furthermore, none of these studies identified deaths that occurred after discharge. The remainder of the studies contained less than 1000 patients and therefore could not provide accurate estimates of mortality trends.

For variceal hemorrhage, the largest study on mortality after hospitalization because of varices (n = 12,281; compared with 14,682 for this study) did not differentiate between hemorrhage and nonhemorrhage admissions.^26^ The next largest study (n = 1475) compared variceal hemorrhage mortality between control groups in randomized trials 1960–2000 and showed a similar reduction in mortality.^27^ However, these control groups were from different geographical populations with different study exclusion criteria. Comparisons were therefore susceptible to selection bias. Other studies of trends in variceal hemorrhage mortality contained less than 1000 patients.

The other finding of note in our study in relation to variceal hemorrhage is the small proportion of overall hemorrhages that they represent. In the context of the increasing burden of liver disease^28^ and an apparent increase in variceal hemorrhage in the recent BSG audit,^8^ a higher proportion might have been expected. Our finding, however, was similar to that from the 1993 BSG audit (4%) and to other studies.^9,29^ It is possible that some of the variceal hemorrhages in our study may have been incorrectly coded to esophageal hemorrhage, but a sensitivity analysis, assuming the most likely misclassification of all esophageal hemorrhage codes being miscoded variceal bleeds, did not alter the adjusted reduction in mortality.

The previous difficulties in detecting a reduction in mortality might imply that we are reaching the point where mortality becomes unavoidable because of age and comorbidity. However, because the mortality in our study continued to improve right up to the end of the study period, improvements in management would appear to be continuing to have an impact on mortality following gastrointestinal hemorrhage. The reasons for the reduction in mortality we have observed are likely to be complex. There were similar reductions in mortality whether or not an endoscopy was recorded and for all associated diagnoses, implying that endoscopic therapy was not a major contributor to the reduction in mortality. Instead, our data perhaps suggest that improvement in standard nonendoscopic care has led to improved survival, such as the routine administration of intravenous proton pump inhibitor infusions, the routine use of risk scoring, the implementation of standardized clinical guidelines, and the subsequent local auditing of practice.^4,5,30^

In conclusion, contrary to previous smaller studies, we have found an encouraging substantial improvement in mortality following hospital admission for upper gastrointestinal hemorrhage. Our study shows that this is partially obscured by changes in age and comorbidity and that the improvements are less marked in the elderly individuals in a manner not explained by comorbidity. We believe that this improvement reflects the effect of changes in the care of gastrointestinal hemorrhage over the last decade, but it also suggests the need to focus our ongoing attention on the elderly individuals who may not yet have benefited to the maximum possible extent from these changes. The recent demonstration of under-utilization of endoscopic techniques in the United Kingdom, coupled with the fact that other interventions such as use of proton pump inhibitors are more readily available to the admitting physician worldwide, may suggest areas that could be further improved.^4,5,31–33^

## Figures and Tables

**Table 1:** Population Characteristics

	Nonvariceal bleed admissions			Variceal bleed admissions		
	Number of admissions (n)	Percentage of all admissions	28-Day case fatality (%)	Number of admissions (n)	Percentage of all admissions	28-Day case fatality (%)
Year						
1999	51,843	10.3	14.7	1559	10.6	24.6
2000	53,206	10.6	14.8	1592	10.8	25.1
2001	53,268	10.6	14.9	1496	10.2	25.0
2002	53,735	10.7	14.9	1581	10.8	24.2
2003	55,656	11.1	14.7	1619	11.0	23.6
2004	57,450	11.5	14.1	1768	12.0	22.3
2005	59,362	11.8	13.9	1612	11.0	21.7
2006	58,737	11.7	13.7	1736	11.8	20.7
2007	58,214	11.6	13.1	1719	11.7	20.9
Total	501,471	100.0	14.3	14,682	100.0	23.1
Sex						
Male	276,304	55.1	13.3	9565	65.1	23.0
Female	225,167	44.9	15.5	5117	34.9	23.2
Age, *y*						
<30	39,973	8.0	0.5	375	2.6	10.7
30 to 59	135,507	27.0	5.5	8749	59.6	21.2
60 to 79	174,181	34.7	15.1	4688	31.9	25.9
≥80	151,810	30.3	24.8	870	5.9	31.3
Charlson index						
No comorbidity	229,941	45.9	6.8	9825	66.9	21.6
Single comorbidity	150,004	29.9	13.6	3832	26.1	25.2
Multiple or serious comorbidity	121,526	24.2	29.2	1025	7.0	29.5
Acute hemorrhage on admission or inpatient						
Acute hemorrhage on admission	295,887	59.0	10.5	10,176	69.3	20.1
Inpatient bleed	205,584	41.0	19.7	4506	30.7	29.8

NOTE. Linked HES/ONS mortality records are currently provided on a provisional basis. An issue has arisen whereby a small number of mortality records may have been incorrectly rejected. The algorithm that links HES to ONS mortality is currently being amended to rectify this issue, which affects approximately 1000 mortality records or about 0.02% of the total.

**Table 2:** Logistic Regression Model Predicting 28-Day Mortality

Nonvariceal hemorrhage				Variceal hemorrhage		
	Unadjusted odds ratio	Adjusted odds ratio^a^	95% Confidence interval	Unadjusted odds ratio	Adjusted odds ratio^a^	95% Confidence interval
Year of presentation						
1999	1.00	1.00			1.00	1.00
2000	1.00	0.98	0.94–1.01	1.02	1.02	0.87–1.20
2001	1.01	0.97	0.93–1.00	1.02	1.02	0.86–1.20
2002	1.01	0.95	0.92–0.99	0.98	0.98	0.83–1.15
2003	0.99	0.94	0.90–0.97	0.94	0.95	0.80–1.11
2004	0.95	0.90	0.86–0.93	0.88	0.88	0.75–1.03
2005	0.93	0.89	0.86–0.92	0.85	0.83	0.70–0.98
2006	0.92	0.85	0.82–0.88	0.80	0.79	0.67–0.94
2007	0.87	0.80	0.77–0.83	0.81	0.80	0.67–0.94
Age, *y*						
<30	1.00	1.00		1.00	1.00	
30–59	10.09	7.22	6.37–8.19	1.93	1.92	1.44–2.55
60–79	30.04	16.80	14.84–19.02	2.51	2.37	1.77–3.17
≥80	55.62	34.14	30.15–38.65	3.26	3.05	2.22–4.20
Sex						
Male	1.00	1.00		1.00	1.00	
Female	1.20	1.01	0.99–1.03	1.01	0.96	0.88–1.04
Charlson index						
No comorbidity	1.00	1.00		1.00	1.00	
Single comorbidity	2.16	1.70	1.66–1.74	0.99	1.17	1.07–1.27
Multiple or serious comorbidity	5.64	4.37	4.28–4.47	1.31	1.37	1.18–1.58

NOTE. Linked HES/ONS mortality records are currently provided on a provisional basis. An issue has arisen whereby a small number of mortality records may have been incorrectly rejected. The algorithm that links HES to ONS mortality is currently being amended to rectify this issue, which affects approximately 1000 mortality records or about 0.02% of the total.

a Adjusted for all variables in Table.

**Table 3:** Trends in 28-Day Mortality for Diagnoses Associated With an Upper Gastrointestinal Hemorrhage

Diagnosis associated with upper gastrointestinal hemorrhage	Adjusted odds ratio^a^	95% confidence intervals
Change in mortality for an increment of 1 year^b^		
No specific diagnosis	0.97	0.97–0.98
Gastritis/duodenitis	0.96	0.94–0.98
Mallory–Weiss syndrome	0.96	0.95–0.97
Any peptic ulcer	0.96	0.93–0.99
Gastric ulcer	0.94	0.93–0.95
Duodenal ulcer	0.96	0.95–0.97
Malignancy	0.95	0.95–0.96

NOTE. Linked HES/ONS mortality records are currently provided on a provisional basis. An issue has arisen whereby a small number of mortality records may have been incorrectly rejected. The algorithm that links HES to ONS mortality is currently being amended to rectify this issue, which affects approximately 1000 mortality records or about 0.02% of the total.

a Adjusted for age, sex, and comorbidity by Charlson index.

b Year as a continuous variable.

**Table 4:** Age Stratified Logistic Regression Model Predicting 28-Day Mortality for Nonvariceal Hemorrhage

	Adjusted odds ratio^a^	95% Confidence interval
Change in mortality for an increment of 1 y^b^		
<30 *y*	0.92	0.88–0.97
30–59 *y*	0.97	0.96–0.97
60–79 *y*	0.97	0.96–0.97
≥80 *y*	0.99	0.98–0.99

NOTE. Linked HES/ONS mortality records are currently provided on a provisional basis. An issue has arisen whereby a small number of mortality records may have been incorrectly rejected. The algorithm that links HES to ONS mortality is currently being amended to rectify this issue, which affects approximately 1000 mortality records or about 0.02% of the total.

a Adjusted for comorbidity by Charlson index and sex.

b Year as a continuous variable.

**Table 5:** Age and Comorbidity Stratified Logistic Regression Model Predicting 28-Day Mortality

Age, *y*	Charlson index	Adjusted odds ratio^a^	95% Confidence interval
Change in mortality for an increment of 1 y^b^			
<80			
	0	0.96	0.95–0.97
	1	0.96	0.95–0.97
	2	0.95	0.95–0.96
≥80			
	0	1.00	0.99–1.01
	1	0.99	0.98–0.99
	2	0.98	0.97–0.99

NOTE. Linked HES/ONS mortality records are currently provided on a provisional basis. An issue has arisen whereby a small number of mortality records may have been incorrectly rejected. The algorithm that links HES to ONS mortality is currently being amended to rectify this issue, which affects approximately 1000 mortality records or about 0.02% of the total.

a Adjusted for sex.

b Odds ratio for year as a continuous variable.

## Video Abstract

Video Abstract
